# Transplanted bone marrow stem cells relocate to infarct penumbra and co-express endogenous proliferative and immature neuronal markers in a mouse model of ischemic cerebral stroke

**DOI:** 10.1186/1471-2202-11-138

**Published:** 2010-10-25

**Authors:** Xue-mei Zhang, Fang Du, Dan Yang, Chun-jiang Yu, Xiang-nan Huang, Wei Liu, Jin Fu

**Affiliations:** 1 Department of Neurology, The Second Affiliated Hospital, Harbin Medical University, Harbin, 150086, China; 2 Center of Research and Experiment, The Second Affiliated Hospital, Harbin Medical University, Harbin, 150086, China

## Abstract

**Background:**

Several studies demonstrate that neurogenesis may be induced or activated following vascular insults, which may be important for neuronal regeneration and functional recovery. Understanding the cellular mechanism underlying stroke-associated neurogenesis is of neurobiological as well as neurological/clinical relevance. The present study attempted to explore potential homing and early development of transplanted bone marrow stem cells in mouse forebrain after focal occlusion of the middle cerebral artery, an experimental model of ischemic stroke.

**Results:**

Bone marrow stem cells isolated from donor mice were confirmed by analysis of surface antigen profile, and were pre-labeled with a lipophilic fluorescent dye PKH26, and subsequently transfused into recipient mice with middle cerebral artery coagulation. A large number of PKH26-labeled cells were detected surrounding the infarct site, most of which colocalized with immunolabelings for the proliferating cell nuclear antigen (PCNA) and some also colocalized with the immature neuronal marker doublecortin (DCX) during 1-2 weeks after the bone marrow cells transfusion.

**Conclusions:**

The present study shows that transplanted bone morrow cells largely relocate to the infarct penumbra in ischemic mouse cerebrum. These transplanted bone marrow cells appear to undergo a process of in situ proliferation and develop into putative cortical interneurons during the early phase of experimental vascular injury.

## Background

Cerebral strokes due to ischemic, embolic and hemorrhagic insults are common neurological conditions that cause brain damage and functional loss. Associated with or subsequent to these insults are broad host responses at molecular and cellular levels involving both the neuronal and non-neuronal components of the brain. For instance, stroke-related anatomical/pathological changes may include infiltration of blood cells, angiogenesis and activation/proliferation of glial cells [1-3]. In addition, recent studies indicate that neurogenesis may be induced or activated following vascular insults, which may be important for neuronal regeneration and functional recovery [3-7]. Thus, understanding the cellular mechanism(s) underlying stroke-associated neurogenesis is of neurobiological as well as neurological/medical implications.

One of the key issues related to stroke-induced neurogenesis concerns the origin of cells that may give rise to new neurons. It is currently considered that neurogenesis in the adult brain occurs in restricted areas under physiological conditions, namely the subventricular zone (SVZ) and subgranular zone (SGZ) [8,9]. However, stroke and trauma-induced neurogenesis have been described in broad brain regions/sites than the SVZ/SGZ [10-14]. Of interest, bone marrow cells may differentiate into various types of peripheral cells and likely neurons as well [15-17]. There is evidence from humans and laboratory animals that new neurons in the brain may arise from putative blood-borne cells [18,19]. It appears that putative bone marrow cells may be particularly important for adult neurogenesis following stroke or traumatic brain injury [1,20]. Less is known with regard to the seeding and early phase of proliferation or neuronal differentiation of bone marrow cells in the stroke-injured brain. To address these issues, we isolated bone marrow mononuclear cells (BMMCs) from adult mice, pre-labeled them with a lipophilic red fluorescence dye PKH26 [21], and tracked these cells in vivo following transfusion into mice with middle cerebral coagulation. We detected colocalization around the infarct site of PKH26-labeled cells with the endogenous cell division marker, proliferating cell nuclear antigen (PCNA), and with the immature neuronal marker doublecortin (DCX) [22,23]. These data appear to support that bone marrow cells may be one of the important sources of stem cells involved in neurogenesis following acute cerebral vascular injury, and that transplantation of these cells is of potential clinical utility in the management of stroke.

## Results

### Cytofluorometric characterization of bone marrow cell preparations

**Figure 1:**
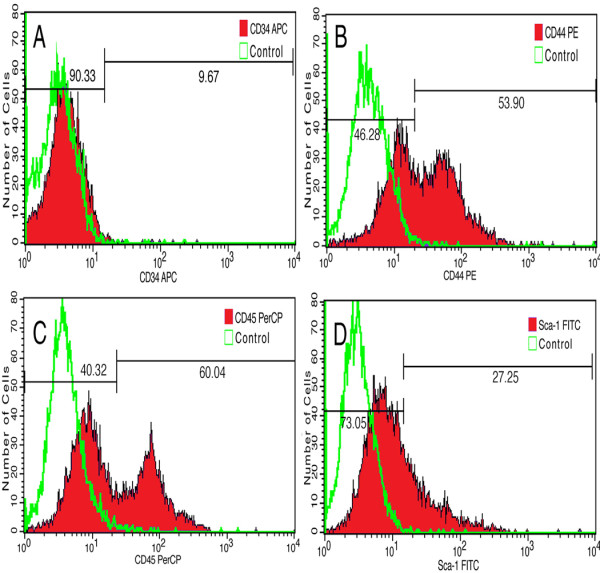
**Fluorescence-activated cell sorting analyses the expression of mesenchymal stem cell markers**. APC-conjugated anti-mouse CD34 reactivity is detected on 9.67% of the isolated cells (A). PE-conjugated anti-mouse/human CD44 reactivity is present on 53.90% of the cells (B). Percp-conjugated anti-mouse CD45 antibody labels 60.04% of the cells (C), and FITC-conjugated anti-mouse LY-6A/EC Sca-1 antibody reacts to 27.25% of the cells (D).

Four fluorescence-activated cell sorting (FACS) analyses were used to determine the expression of various signature antigen markers of mesenchymal stem cells on the isolated cells (Figure 1). In brief, 9.67% of the isolated cells expressed CD34 (Figure 1A); 53.90% expressed CD44 (Figure 1B); 27.25% expressed stem cell marker, Sca-1 (Figure 1C); and 60.04% expressed CD45 (Figure 1D). Taken together, the data implicated that the isolated bone marrow cells exhibited a panel of surface antigens characteristic of the haemopoietic stem cell population [24]. In other words, the bone marrow cells derived from BALB/c mouse long bones appeared to be largely BMMCs according to established evaluation protocols [25,26].

### Distribution of transplanted bone marrow cells around cerebral infarct

**Figure 2:**
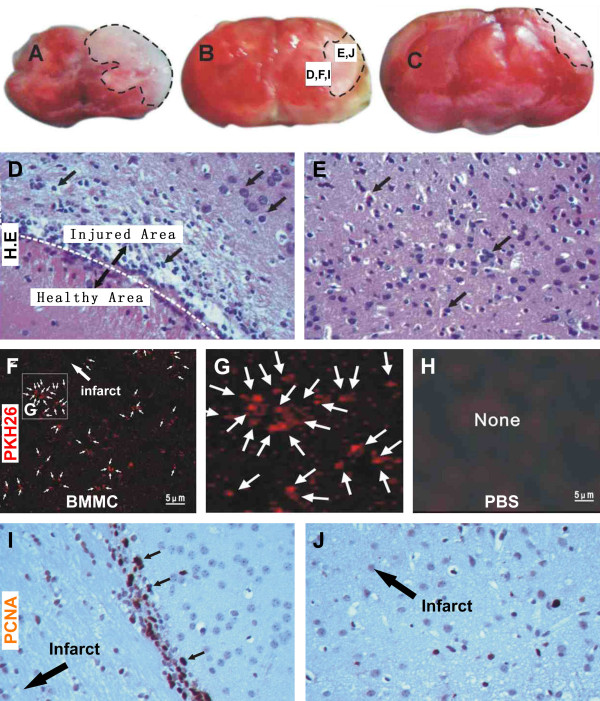
**Localization of transplanted cells and in situ proliferation at the infarct border in experimental model**. (A-C) are images of coronal cerebral slices showing the impact of occlusion 6 hours post-occlusion, with the infarct appearing as pale area marked with broken line. Hematoxylin and eosin (H.E.) stain illustrates loss of relatively large cells and infiltration of small cells at the border of the infarct (D). Paired cellular profiles are seen peripheral to the infarct border or the infarct penumbra (E). At low magnification, a large number of transplanted bone marrow cells pre-labeled by PKH26 (red fluorescence) are present in the infarct penumbra 7 days post lesion (F). The labeled cells are small and may occur in cluster (G). No fluorescent cells exist in the cerebral cortex of mice received vehicle infusion (H). In situ cell division reflected by immunoreactivity of proliferating cell nuclear antigen (PCNA) occurs predominantly at the infarct border (I) and penumbra (J), appearing as brown immunoreactive nuclei in hematoxylin counter-stained section.

In the present study we established focal cerebral ischemia/infarction under visually guided local occlusion of cerebral artery. To confirm the effect of unilateral electric coagulation of the middle cerebral artery, a group of animals were sacrificed 6 hours after the surgery, and their brains were removed without perfusion and were cut in ~3 mm thick coronal slices (Figure 2A-C). Gross appearance of focal ischemia was evident in the operated hemisphere, with a pale infarct area located around the lateral-middle portion of cerebrum. In H.E. stained sections from these brains, signs of tissue necrosis were present in and around the infarct, including vacuolation, shrunken neurons and eosinophilic cytoplasm (Figure 2D, E).

In our pilot studies, we looked PKH26 fluorescent signal 1 to 4 weeks post bone marrow cells transplantation in the ischemic and control brains. Fluorescent cells were detectable in the former group up to ~3 weeks, but tended to fade afterwards (data not shown). In normal control brain, only a very few fluorescent cells were encountered (data not shown), suggesting increased infiltration of blood cells after vascular injury. For these reasons, histological and immunohistochemical analyses were carried out at 1 and 2 weeks post cell transplant. Thus, at the one week surviving time point, a large number of PKH26-labeled cells were found in the ischemic brains, which were located primarily around the border of the infarct area and the peripheral healthy regions in the cortex, or the infarct penumbra (Figure 2F). These cells were small in size and appeared often in group or cluster (Figure 2G). No apparent cellular processes were visible on these cells. In all examined brain sections from animals received PBS as vehicle control, no PKH26-labeled cells were found (Figure 2H).

### Colocalization of transplanted bone marrow cells with PCNA

Immunolabeling for the proliferating cell nuclear antigen (PCNA) was used to detect in situ cell proliferative activity in the ischemic mouse cortex. A large number of PCNA immunoreactive nuclei were present at the junction of the infarct site to the peripheral, relatively healthy, areas at 1 (Figure 2I) and 2 (not shown) weeks post cerebral artery occlusion. Fewer labeled cells were also seen interior to this border (i.e., towards the infarct center) (Figure 2I, J). PCNA immunoreactive profiles were all cell nuclei as confirmed by haematoxylin counterstain, and they exhibited variable labeling intensity and sizes. In addition, some of PCNA labeled nuclei appeared to occur in pair (Figure 2I, small arrows).

**Figure 3:**
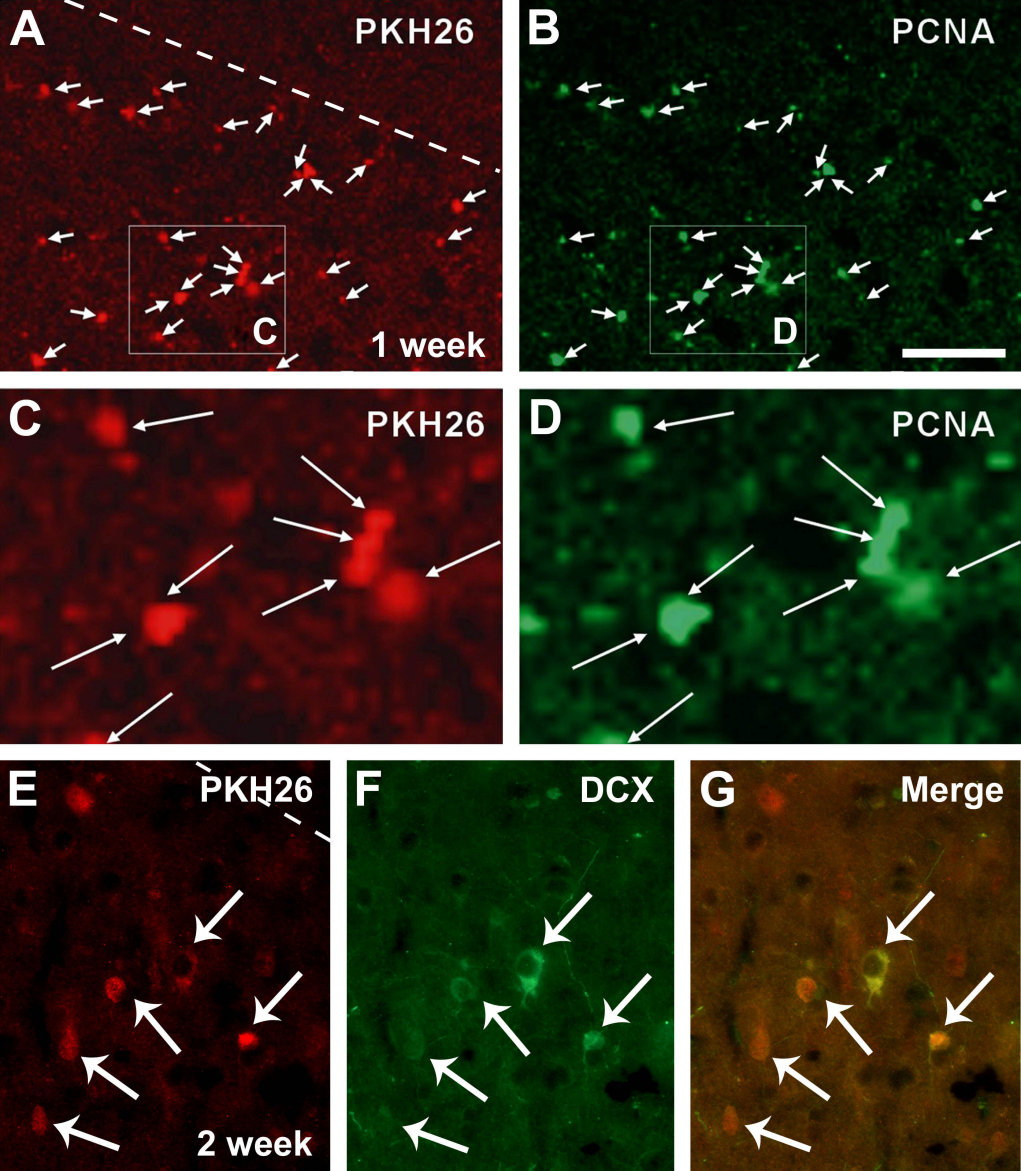
**Identification of PKH26-labeled cells with proliferating cell nuclear antigen and doublecortin after transplantation**. Colocalization of PKH26-labeled bone marrow cells with proliferating cell nuclear antigen (PCNA) (A-D) and doublecortin (DCX) (E-G) around the infarct penumbra 7 and 14 days following transplantation. PKH26 and PCNA double-labeled cells are small and occur in pair or small cluster (C, D). PKH26 and DCX double-labeled cells have round or oval somata with visible neuronal processes. PKH26 fluorescence appears weaker in these double-labeled cells relative to those seen at 7 days post cell transplantation. Scale bar (in B) = 200 μm for A, B and 50 μm for C-G.

In order to explore if transplanted bone marrow cells might be a part of the proliferating cells seen around the infarct areas, we carried out double labeling analysis for colocalization of PCNA (visualized with Alexa-488 conjugated secondary antibody) and PKH26. In the brains examined 1 week after bone marrow cell transplant, most PKH26-labeled cells were found to colocalize with PCNA (Figure 3A-D) immunoreactivity. Similar to the above described distribution of cells labeled by PKH26 or PCNA, the double-labeled cells occurred predominantly around the infarct penumbra. Many of the double-labeled cells appeared in pair or in small cluster, optimal for a morphochemical pattern of actively proliferating cells (Figure 3C, D).

### Colocalization of transplanted bone marrow cells with doublecortin

Doublecortin (DCX) is an immature neuronal marker expressed by newborn neurons in the subventricular and subgranular zones in the adult mammalian brain [23]. DCX labeling can be used to detect recently-generated immature neurons after traumatic injury ischemia in the brain including the cerebral cortex [3,14]. We used this marker to explore if transplanted bone marrow cells might differentiate toward neuronal phenotype in the ischemic mouse cerebral cortex. In double fluorescent preparations, we did not detect clear colocalization of DCX among PKH26-labeled cells at 1 week post bone marrow cell transplant (data not shown). However, in sections from animals survived for two weeks, some PKH26-labeled cells appeared to colocalize with DCX. These double-labeled cells were again found around the infarct penumbra. Some of the double-labeled cells had relatively large somal size with visible dendrite-like processes, suggestive of a neuronal morphology (Figure 3F). Among the colocalized cells, those with larger somata expressed weaker PKH26 fluorescence relative to the ones with smaller size (Figure 3E). Thus, there appeared to be a trend of reduction or dilution of PKH26 labeling with cell growth. Overall, the morphology of the double-labeled cells resembled interneurons rather large pyramidal neurons in the cerebral cortex [14]. In longer surviving time point (3-4 weeks), we failed to detect double-labeled cells likely because of the fading of PKH26 fluorescence with time (not shown).

## Discussion

Significant advance has been made during recent years in the field of stem cell therapy [27,28]. Because pluripotent bone marrow stem cells can be obtained from a given individual and transplanted in a form of autograft following potential in vitro expansion, these cells are considered to be of particular medical use for tissue/organ repair [15,16,29]. In many peripheral organs, transplantation of bone marrows stromal cells appear to facilitate tissue regeneration and improve functional recovery following acute injury and even under certain chronic degenerative conditions [17,26,30-34]. Bone marrow stem cells are also considered to be useful in the treatment of acute brain injury and certain degenerative neurological disorders [17,28]. In animal models of cerebral stroke and traumatic injury bone marrow cell therapy show beneficial effects, as evidenced by improved histological outcome and neurological performance [35-37].

Evidence exists in supportive of an involvement of putative blood-borne cells, likely stromal stem cells, in neurogenesis in mature mammalian brain. In humans as well as animals, mature neurons containing Y-chromosome are detected in the cerebrum of females that have received blood from males [18,19]. It appears that bone marrow stem cells might contribute to adult neurogenesis more significantly following brain injury. A number of previous studies report that transplanted bone marrow cells pre-labeled with BrdU or retrovirus colocalize with mature neuronal (also glial and endothelial) markers, such as MAP-2 and NeuN, in the cerebrum following ischemic injury [35,38-40]. However, few studies have investigated the early stage of development or transdifferentiation of the transplanted bone marrow cells in injured host brain.

In the present study, bone marrow cells are isolated from adult mouse long bone, and are subsequently characterized using a panel of haemopoietic stem cell markers. The isolated bone marrow cells express CD34 (9.67%), CD44 (53.90%), Sca-1 (27.25%) and CD45 (60.04%), therefore confirming a phenotype of mesenchymal stem cells. Then, a lipophilic marker PKH26 is used to pre-label the isolated bone marrow stem cells, which allow us to track a large population of these cells in vivo for a few weeks following transplantation. In the ischemic mouse cerebral cortex, most of the PKH26-labeled bone marrow stem cells are localized to the periphery or the penumbra of ischemic infarct. This distribution pattern suggests that the cells are likely released from the blood vessels of the neighboring healthy cortex, or alternatively that the cells survive better around the infarct penumbra because of a relatively intact blood supply. Of note, we observe a considerable amount of proliferative activity in the same peri-infarct region, as reflected by PCNA expression. Moreover, there exists a great degree of colocalization between PCNA and PKH26 around the infarct penumbra. Therefore, the transplanted bone marrow stem cells might undergo in situ proliferation after they have seeded in the ischemic cerebral parenchyma.

Importantly, by two weeks post transfusion many PKH26-labeled cells in the peri-infarct region colocalize with DCX, a marker of immature neurons [23]. The double-labeled cells are relatively small, have round or oval somata, and develop dendrite-like processes. The morphology of these double-labeled cells is more reminiscent of cortical interneurons rather than pyramidal neurons. A previous study also shows that bone-marrow cell derived neurons exhibit stellate, ramified or triangular morphologies [39]. Another study demonstrates that newborn neurons in ischemic rat cortex co-express various markers of cortical interneurons [14]. Thus, our findings are consistent with a notion that the transdifferentiated neurons from bone-marrow cells in the injured cerebrum are morphologically resembled cortical interneurons.

## Conclusions

In summary, following in vitro labeling of a large number of cells with the lipophilic marker PKH26, the present study demonstrates that transplanted bone morrow cells relocate to and reside mostly around the infarct penumbra in a mouse model of ischemic cerebral stroke. These transplanted bone marrow cells might undergo certain extent of in situ proliferation, and they appear to transdifferentiate into putative cortical neurons during the first few weeks of experimental vascular injury.

## Methods

### Experimental animals

BALB/c mice at 8 to 10 weeks of age, either sex, weighing 20 to 22 grams, were purchased from the Animal Center of Second Affiliated Hospital of Harbin Medical University. Animals were housed at constant temperature and humidity, with a 12/12 hr light/dark illumination cycle and free access to food chow and water. All experimental procedures used in the present study were approved by the Harbin Medical University Administrative Panel on Laboratory Animal Care, which is compatible with the NIH guidelines for use and care of laboratory animals.

### Reagents

The primary antibodies and detecting reagents included mouse anti-PCNA antibody (BM0104, Boster company, Santa Cruz Biotechnology), goat-anti-doublecortin (SC-8006, Boster company, Santa Cruz Biotechnology), Alexa Fluor^® ^488 and 594 conjugated donkey anti-mouse and anti-goat IgGs (Invitrogen, Carlsbad, CA). The fluorescent dye PKH26 was obtained from Sigma-Aldrich (MINI26, Saint louis, Missouri, USA). All fluorescent cytoflow markers were purchased from Biolegend (San Diego, USA), including (FITC rat IgG 2 Isotype ctrl. Clone: RT K 2758 (Cat. No. 400505), APC rat IgG 2 Isotype ctrl. Clone: RT K 2758 (400511), Percp rat IgG 2b Isotype ctrl. Clone: RT K 2758 (118419), PE rat IgG 2b Isotype ctrl. Clone: RT K 2758 (119316), APC anti-mouse CD34 Clone: MEC 14.7 (119309), FITC anti-mouse LY-6A/EC Sca-1 Clone: E13-16.1.7 (122505), PE anti-mouse/human CD44 Clone: IM 7 (103007), Percp anti-mouse CD45 Clone: 30-F11 (103129).

### Isolation and lipophilic fluorescent labeling of bone marrow cells

BALB/c mice were anesthetized with sodium pentobarbital (100 mg/kg, i.p.). Femoral bones were removed under sterile condition, with the medullary cavity bathed by heparin (50 U/mL) in normal saline [41,42]. Bone marrow was aspirated and suspended in a lymphocyte isolation medium, followed by centrifuge at 2000 rpm for 20 minutes. The cell pellets were subsequently diluted with DMEM/F12 medium (DMEM/F12, 15% FBS, 100000 U/L penicillin, pH = 7.4) to yield a density of more than 5×10^7 ^cells/mL. The cells were then labeled with the red-fluorescent lipophilic tracer PKH26 according to the manufacturer's instruction [41]. The density of PKH26-labeled cell suspension was adjusted to approximately 3×10^7 ^cells/mL. Cells with viability greater than 95% as measured by trypan blue exclusion were used for the subsequent transplant studies.

### Focal cerebral ischemia and bone marrow cell transfusion

An acute ischemic stroke model was established by coagulation of the middle cerebral artery in experimental mice[2]. In brief, mice were anesthetized with pentobarbital (50 mg/kg, i.p.), and a craniotomy was carried out by maintaining the animals over a heating pad (with rectal temperature at 36.5 to 37.5°C). The left middle cerebral artery (MCA) was exposed, and occluded with a short period of coagulation using a general metallic heat applicator. The cranial hole was sealed with dental cement, and skin sutured before the mice were returned to their cages. After the brain surgery, the experimental animals were infused via tail vein either with PKH26 pre-labeled bone marrow cells (n = 16) or phosphate buffered solution (PBS) as control (n = 16). Animals were allowed to survive for 6 hours (n = 4), and 7 (n = 4), 14 (n = 4) and 21 (n = 4) days post bone marrow cell transplantation.

### Fluorescence-activated cell sorting analysis

To evaluate the purity of the isolated bone marrow cells as putative bone marrow mononuclear cells (BMMCs) or bone marrow stem cells, part of the cell pellet was subject to fluorescence-activated cell sorting (FACS) analysis for the expression of various signature antigen markers of mesenchymal stem cells. Thus, approximately 1×10^6 ^cells were incubated in 2% fetal bovine serum in PBS at 4°C for 30 minutes with 1 μl of monoclonal antibody specific for CD34, CD44, CD45, Sca-1 (all from BioLegend 11080 Roselle Street, San Diego, CA 92121). Negative control was processed by incubating the cells in buffer without primary antibodies. The immunofluorescent signal was analyzed using the FACS Calibur with CellQuest software (Becton Dickinson, USA).

### Tissue preparation

Except for the short time surviving group (6 hrs post surgery), all other animals were perfused transcardially with 4% paraformaldehyde (Sigma-Aldrich, St. Louis, USA) in 0.01 M phosphate-buffered saline (pH 7.4, PBS) under overdose anesthesia (sodium pentobarbital 100 mg/kg, i.p.). Brains were removed from the skull, postfixed in the perfusion fixative overnight, and cryoprotected in 30% sucrose at 4°C. Brains were cut at the coronal plane in a cryostat at 6 μm, and sections passing through the infarct areas were collected alternatively (every 20 sections) by thaw-mounting on gelatin-coated microslides. Sections were stored at -70°C for a few weeks before histological (H.E stain) and immunohistochemical examinations.

### Immunohistochemistry

For immunolabeling with the peroxidase method, sections were first treated 1% H_2_O_2 _in PBS for 30 minutes, and then pre-incubated in 5% normal horse or rabbit serum in PBS with 0.3% Triton X-100 for 1 hour. Subsequently, sections were incubated with mouse anti-PCNA (1:400) or goat anti-DCX (1:100) antibodies in PBS containing the blocking serum and Triton X-100 at 4°C overnight. Sections were further reacted with biotinylated horse anti-mouse or rabbit anti-goat secondary antibodies at 1:400 (BM0104 Boster company, Santa Cruz Biotechnology, CA) for 2 hours, and finally with the avidin-biotin complex (ABC) reagents (1:400) (Vector Laboratories, Burlingame, CA) for another 1 hour. Immunoreation product was visualized in 0.003% H_2_O_2_, 0.05% diaminobenzidine (DAB; Sigma, St, Louis, MO). Three 10-minute washes were used between all incubations. Sections were allowed to air-dry, dehydrated through ascending ethanol, cleared with xylene and coverslipped. Some of the above immunohistochemically prepared sections were counterstained with haematoxylin before dehydration.

To determine the colocalization of PCNA and DCX expression with transplanted BMMCs, additional sections were subject to double fluorescent labeling for PCNA or DCX with PKH26. Sections were incubated in PBS containing 5% normal donkey serum and mouse anti-PCNA antibody (1:400) or goat anti-DCX antibody (1:200) at 4°C overnight, followed by a 2 hours reaction with Alexa Fluor^® ^488 conjugated donkey anti-mouse or goat IgGs (1:200, A21206 Invitrogen, Carlsbad, CA). Sections were then washed and mounted with anti-fading medium before microscopic examination. Sections from animals received tail vein infusion of the PBS vehicle were processed in parallel as negative control for fluorescent labeling.

## Authors' contributions

XMZ participated in the design of the study, making the animal model, carried out the immunohistochemical studies and drafted the manuscript. FD carried out the animal model and bone marrow cell transfusion. XNH and WL helped tissue processing and acquisition of data. DY participated in the fluorescence-activated cell sorting analysis. CJY performed the statistical analysis. JF supervised and coordinated the study, and finalized the manuscript. All authors read and approved the final manuscript.
